# Effect of Salt Reduction Interventions in Lowering Blood Pressure and Salt Intake in Zhejiang Province, China, 2017–2021: A Randomized Controlled Trial

**DOI:** 10.3390/nu17050893

**Published:** 2025-03-03

**Authors:** Xiaofu Du, Ying Zhu, Jing Guo, Xiangyu Chen, Jie Zhang, Feng Lu, Chunxiao Xu, Mingbin Liang, Meng Wang, Jieming Zhong, Yuanyuan Li

**Affiliations:** 1 Key Laboratory of Environment and Health, Ministry of Education & Ministry of Environmental Protection, and State Key Laboratory of Environmental Health, School of Public Health, Tongji Medical College, Huazhong University of Science and Technology, Wuhan 430030, China; xfdu@cdc.zj.cn; 2 Department of Chronic Disease Prevention and Control, Zhejiang Provincial Center for Disease Control and Prevention, Hangzhou 310051, China; xychen@cdc.zj.cn (X.C.); jiezhang@cdc.zj.cn (J.Z.); flu@cdc.zj.cn (F.L.); chxxu@cdc.zj.cn (C.X.); mbliang@cdc.zj.cn (M.L.); mwang@cdc.zj.cn (M.W.); 3 Jiashan County Center for Disease Control and Prevention, Jiaxing 314199, China; zhuying147@sina.com; 4 Department of Epidemiology, School of Public Health, Zhejiang Chinese Medical University, Hangzhou 310053, China; guojingzju@zju.edu.cn

**Keywords:** community intervention, salt reduction, effect evaluation, sodium, blood pressure

## Abstract

Background: Addressing high-salt diets in China through interventions can significantly reduce blood pressure (BP) and the associated health risks. Objective: This study aims to evaluate the effectiveness of a comprehensive salt reduction intervention implemented across counties in Zhejiang Province, focusing on system establishment, extensive publicity, and targeted population interventions. Methods: The Salt Reduction and Hypertension Prevention Project was initiated in Zhejiang Province. Cross-sectional surveys were conducted before the intervention and after. The research commenced in 2017 with a baseline survey involving 7512 participants from five counties. Four counties were randomly selected for the intervention, implementing a multifaceted salt reduction strategy, while one county served as a reference without any intervention. The primary outcomes measured were changes in BP and 24 h urinary sodium and potassium excretion. Results: Following the intervention, 24 h urinary potassium excretion experienced a significant increase, rising from 1441.3 (SD 681.9) to 1676.9 (SD 931.4) mg per day, *p* < 0.001. Utilizing a linear mixed-effects model, the adjusted net difference in urinary sodium changes was calculated to be 394.1 mg per day (95% CI, 133.2 to 655.0) (*p* = 0.003). There was a notable reduction in systolic blood pressure (SBP) from 131.2 (SD 19.2) to 129.8 mmHg (SD 18.0), and diastolic blood pressure (DBP) also decreased from 80.8 (SD 10.8) to 78.9 mmHg (SD 10.2), *p* < 0.001. The adjusted net differences for SBP and DBP between the intervention and reference groups were 1.3 (95%CI, 0.5 to 2.1) and 1.4 mmHg (95%CI, 0.9 to 2.0), respectively, *p* < 0.001. Conclusions: The findings indicate that a multi-sectoral approach, combined with extensive public awareness initiatives and precisely targeted interventions, can significantly increase urinary potassium excretion and reduce sodium and blood pressure.

## 1. Background

Hypertension stands as one of the leading preventable global risk factors contributing to cardiovascular disease and premature mortality [1]. In China, hypertension and prehypertension are highly prevalent and rising. A national survey (2012–2015) found 292 million adults (27.8% of the population) had hypertension, and 435.3 million had prehypertension [2]. The causality and the linear relationship between sodium intake and the risks of elevated blood pressure (BP) and cardiovascular disease have been firmly established. The Global Burden of Disease Study estimated that dietary sodium intake contributed to approximately 1.8 million deaths globally in 2019, primarily due to cardiovascular diseases. Reducing sodium intake is not only crucial for managing hypertension, but also for reducing the incidence and prevalence of cardiovascular diseases, thereby lowering mortality and improving overall population health. With an average daily salt intake of 9.3 g, the Chinese population consumes nearly double the amount recommended (less than 5 g per day) by the World Health Organization (WHO), highlighting the pressing need for significant dietary changes to reduce hypertension risk. Each nation has developed its own strategies for sodium reduction [3]. In 2013, the WHO set an ambitious global target aimed at achieving a 30% reduction in the mean population sodium intake by 2025. Subsequently, in 2017, the State Council of China pledged to reduce residents’ salt intake by 20% by the year 2030. In China, where salt consumption is among the highest globally, altering individuals’ dietary habits poses a significant challenge, making the reduction in salt intake a formidable task [4]. There is a close relationship between salt intake and 24 h urinary sodium excretion. The 24 h urinary sodium excretion is considered the “gold standard” for assessing salt intake, as it directly reflects the actual absorption and excretion of sodium in the body. The entrenched long-term preference for high-salt food among residents, coupled with the unsustainable approaches to salt reduction employed by various organizations, has resulted in only modest progress in curbing sodium intake.

Since 2017, the Salt Reduction and Hypertension Prevention Project (SRHPP) in Zhejiang Province, China, has been designing and executing a suite of initiatives under the auspices of the China Center for Disease Control and Prevention (CDC). These include public health education, a salt reduction campaign with extensive media outreach, community-based initiatives targeting hypertensive patients and high-risk groups, and pilot programs in schools, institutions, and food service settings. These intervention strategies are exclusively rolled out in the designated intervention counties and districts, with a reference district/county set up for comparison. Consequently, the SRHPP’s research is focused on establishing a county-level randomized controlled trial that develops systematic intervention measures and protocols for salt reduction. These are intended to leverage existing operational models and platforms, with the potential for national or even global dissemination. Both baseline and follow-up surveys are being conducted within the project areas with the goal of assessing the impact of the salt reduction measures. This study seeks to determine whether the implemented interventions correlate with a reduction in both sodium intake and BP among the populace.

## 2. Methods

### 2.1. Study Setting and Overall Design

The SRHPP is crafted as a cluster randomized controlled trial (RCT), initiated in January 2017 and concluded by the end of 2021. The study encompasses a baseline cross-sectional survey and a terminal effectiveness evaluation across all sites, with equitable representation from both intervention and reference clusters. During the interval between these two surveys, a comprehensive salt reduction intervention was implemented exclusively in the intervention group, spanning a duration of 24 months. This study hypothesizes that extensive salt reduction interventions will lower participants’ daily salt intake to WHO-recommended levels, reducing their BP.

To ensure representativeness, five counties (districts) were selected from Zhejiang Province’s 90 counties, covering different regions. Then, using proportional probability sampling, five towns (rural) or streets (urban) were chosen from each county based on household registration type. Next, three villages or communities were selected from each sampled township or street. The project covered 75 villages or communities in total. From each, 100 adults aged 18–69 were randomly selected. In 2017, 7512 adults were included, with 1496 selected for 24 h urine tests [5]. In 2021, there were 6010 participants for exams/interviews and 1300 for urine data collection.

**Figure 1:**
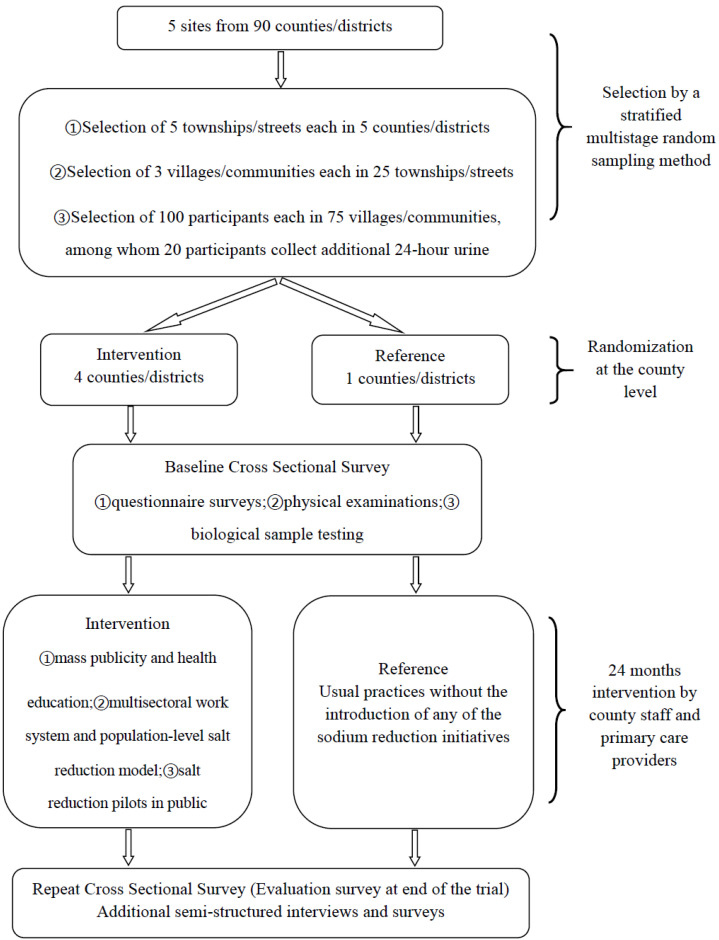
Study design of SRHPP trial.

In accordance with the principles of randomized parallel controlled trials, five pilot sites were allocated by random sequence method to either the intervention group or the reference group with a ratio of 4:1. Absolute geographical separation ensured minimal cross-contamination between the sites. Ultimately, Yiwu City, Haining City, Taishun County, and Yinzhou District of Ningbo City were designated as intervention areas, while the Liandu District of Lishui City served as the reference area with no intervention. Randomization took place following the collection of baseline data, ensuring that those involved in recruitment and baseline surveys, as well as local investigators, remained unaware of the group assignments until the commencement of the intervention. The intervention measures were spearheaded by county investigators with the backing of local health institutions, and were implemented at the county and town levels. The intervention spanned a period of 24 months. The reference group maintained its routine practices without the introduction of any sodium reduction initiatives, resulting in minimal exposure to efforts aimed at achieving individual or population-wide salt reduction. The effectiveness of the intervention was assessed after its completion (i.e., endpoint assessment). The study was approved by the Ethics Review Committee of Zhejiang Provincial CDC, and written consent was obtained from all participants. A detailed study flowchart is depicted in Figure 1.

The preintervention and postintervention surveys were granted approval by the Ethics Committees of the Zhejiang Provincial Center for Disease Control and Prevention. The trial’s methodology and findings are reported in compliance with the CONSORT (Consolidated Standards of Reporting Trials) guidelines, as illustrated in Supplemental Figure S1. The trial has been duly registered with the China Clinical Trial Registry (ChiCTR2000033349). To date, the project has successfully concluded the baseline survey and intervention phase of the trial, adhering to the content as registered. Subsequently, the final effectiveness evaluation has been conducted in alignment with the established protocol.

All participants were invited to participate in confidential in-person interviews and physical examinations conducted at the central office of each community or village. The survey system was designed to gather comprehensive data, encompassing demographic characteristics, general health status, knowledge, attitudes, and behaviors (KABs) pertaining to dietary sodium intake, as well as physical measurement indicators such as BP and 24 h urinary sodium (24-hUNa) and potassium (24-hUKa) excretion [6]. All procedures, including questionnaire surveys, physical examinations, and biological specimen testing, were performed in strict adherence to pertinent guidelines and regulations. The interviews were conducted by trained interviewers who ensured a consistent and standardized approach throughout the process. The determination of the sample size, along with comprehensive details about participant recruitment, BP measurement protocols, urine sample collection, and analytical processes, and the enforcement of quality control measures, was meticulously detailed in the relevant literature [7]. Additionally, a questionnaire pertaining to knowledge, attitudes, and behaviors regarding salt intake is presented in Supplemental Table S1.

### 2.2. Intervention

The SRHPP adopted a multi-sectoral approach, collaborating with stakeholders like the education sector, trade unions, women’s associations, propaganda departments, and food and drug regulators to form a coordinated work pattern, establishing long-term systems for salt reduction.

The program launched a broad public awareness campaign on salt reduction, covering health impacts of salt, risks of excessive intake, salt sources, low-sodium alternatives, salt-limiting strategies, and debunking salt-related myths. It used diverse media, including TV, radio, print, Weibo, WeChat, and Tiktok, to spread knowledge and skills, also utilizing public transportation, stations, and digital screens.

A comprehensive salt reduction model was established, with communities in intervention areas providing low-salt diet information through billboards, posters, illustrations, and leaflets. They organized educational seminars and community activities via village or neighborhood committees, and provided residents with salt-restricting spoons and personal guidance on monitoring and reducing household salt intake.

The project also disseminated salt reduction information through posters, leaflets, banners, and videos in community health services under township hospitals and village clinics. Medical staff in intervention areas were trained on salt and hypertension, and primary healthcare institutions targeted pre-hypertensive individuals and those with hypertension or vascular diseases. They implemented interventions including follow-up, monitoring, and dietary advice, offering tailored guidance on dietary habits and hypertension-related risk factors, and distributed low-salt diet manuals to residents’ families.

### 2.3. Outcomes and Outcome Assessment

The primary outcomes measured were the changes in BP and 24-hUNa and 24-hUKa excretion levels from 2017 to 2021. Secondary outcomes included changes in KABs related to salt consumption. Both the intervention and reference groups underwent parallel evaluations using identical methodologies. Throughout the trial, from the initial baseline to its conclusion, comparisons were made between the intervention and reference groups to assess differences in the changes in 24-hUNa and 24-hUKa excretion, BP, and KABs related to salt. These comparisons aimed to evaluate the impact of the intervention on these key indicators.

### 2.4. Statistical Analysis

We utilized multivariable regression models to assess the variances in 24-hUNa and 24-hUKa levels, the sodium-to-potassium ratio, and blood BP between the surveys conducted in 2017 and 2020. The net differences between the intervention and reference groups were evaluated using a linear mixed-effects model [8]. To determine the difference in the prevalence of correct KABs regarding salt intake and hypertension management between the two groups, we employed a generalized linear mixed-effects model with two-sided tests at a 5% significance level. In a subsequent analysis, we adjusted for significant covariates including age, sex, education level, history of antihypertensive medication use, baseline body mass index, and baseline systolic (or diastolic) blood pressure (SBP, DBP), as well as 24-hUNa and 24-hUKa excretion levels. All statistical tests were two-tailed, with a *p*-value of less than 0.05 deemed to indicate statistical significance. The analyses were conducted using SPSS version 26.0 (IBM Corporation, Armonk, NY, USA).

## 3. Results

### 3.1. Descriptive Statistics

In the baseline survey conducted in 2017, a cohort of 7512 participants had an average age of 44.8 years (standard deviation [SD], 14.0), with 3745 individuals (49.3%) identifying as male. The average SBP recorded was 129.5 mmHg (SD 19.0), while the average DBP was 80.4 mmHg (SD 10.9). Within the intervention group, the average age was marginally higher, at 46.5 years (SD 13.7), with a gender distribution that was evenly split at 50.0% male. This group’s average SBP was 131.2 mmHg (SD 19.2), and the average DBP was 80.8 mmHg (SD 10.8). Among the 1496 participants who provided complete 24 h urine samples, the overall average 24-hUNa level was measured at 3849.5 mg per day (SD 1661.1), and the 24-hUKa level averaged 1491.1 mg per day (SD 710.9). For the intervention group specifically, the average 24-hUNa level was slightly lower at 3701.0 mg per day (SD 1641.8), with the 24-hUKa level being 1441.3 mg per day (SD 681.9).

The baseline characteristics of both the intervention and reference groups were comparable. Following the intervention—during which the reference group did not adopt any salt reduction strategies—there was a notable decrease in the intervention group’s SBP, from 131.2 (SD 19.2) to 129.8 mmHg (SD 18.0), and in DBP, which decreased from 80.8 (SD 10.8) to 78.9 mmHg (SD 10.2), *p* < 0.001. Furthermore, the 24-hUNa levels in the intervention group dropped from 3701.0 (SD 1641.8) to 3615.1 mg per day (SD 1788.9), *p* = 0.24, while the 24-hUKa levels rose from 1441.3 (SD 681.9) to 1676.9 (SD 931.4) mg per day, *p* < 0.001. Within the reference group, SBP saw a significant increase, while DBP showed a minor decline without statistical significance (refer to Table 1 for details).

### 3.2. Changes in SBP, DBP, Sodium, and Potassium Excretion

Mean SBP and DBP in the intervention group showed a decrease of 1.4 mmHg (95% confidence interval [CI] 0.9 to 1.8) and 1.8 mmHg (95%CI, 1.5 to 2.1), respectively, from the baseline to the final assessment, *p* < 0.001. In contrast, the reference group experienced a significant increase of 0.9 mmHg (0.0 to 1.8) in SBP and a non-significant decrease of 0.2 mmHg (−0.4 to 0.8) in DBP. Using a linear mixed-effects model, the adjusted net differences for SBP and DBP between the intervention and reference groups were 1.3 (95%CI, 0.5 to 2.1) and 1.4 mmHg (95%CI, 0.9 to 2.0), respectively, *p* < 0.001. Using a linear mixed-effects model, the adjusted net difference in urinary sodium changes between the intervention and reference groups was 394.1 mg per day (95%CI, 133.2 to 655.0), *p* = 0.003 (as detailed in Table 2).

This study conducted stratified analyses based on blood pressure categories, including normotension, prehypertension, and hypertension, as detailed in Supplemental Table S2. It was observed that the most significant changes in SBP and DBP were noted in hypertensive patients, with adjusted net differences between the intervention and reference groups being 2.0 (95%CI, 0.9 to 3.1) and 2.2 mmHg (95%CI, 1.2 to 3.2), respectively, following the intervention. The primary alteration in 24-hUNa excretion was observed among individuals with normal blood pressure, with a decrease of 307.4 mg per day (95%CI, 14.3 to 600.5) postintervention. The adjusted net difference between the two groups was 701.4 mg per day (95%CI, 233.8 to 1168.9). Changes in 24-hUKa excretion were predominantly seen in participants with normal blood pressure and those with hypertension, with adjusted net differences of 244.6 mg per day (95%CI, 3.1 to 486.0) and 246.9 mg per day (95%CI, 30.8 to 463.0), respectively.

### 3.3. Changes in Knowledge, Attitudes, and Behaviors Associated with Sodium Reduction

KABs in the intervention group saw a significant increase postintervention, with the differences being statistically significant, *p* < 0.001. In the reference group, only a few differences reached statistical significance. The discrepancies between the intervention and reference group was predominantly positive, signifying that the intervention group exhibited a higher growth rate in KABs compared to the reference group. A larger net difference indicates a more pronounced intervention effect. The generalized linear mixed model results demonstrate that participants in the intervention group had a higher likelihood of improving their KABs relative to the reference group, with odds ratios ranging from 1.12 to 1.74, all *p* < 0.05 (as shown in Table 3).

## 4. Discussion

In line with the WHO’s global target for sodium reduction, all member nations have implemented national strategies and initiatives to decrease salt consumption. Countries like the United States, New Zealand, and Thailand have established concrete goals for sodium reduction [9,10,11,12]. In China, salt intake decreased from 10.5g in 2012 to 9.3g in 2018 [13]. Shandong Province’s salt reduction initiative led to a 24.8% drop in sodium intake over five years, easing the rise in SBP and DBP [14]. Consequently, the upward trend in both SBP and DBP has been mitigated and reduced. Unlike developed countries where most sodium comes from processed foods, 75.8% of China’s salt intake is from household cooking [15]. Considering the characteristically high dietary salt intake in China, a comprehensive suite of preventive measures should be rigorously implemented to restrict salt consumption [16]. The SHRPP research emphasizes a family-based salt reduction approach, including distributing salt-restricting spoons and low-sodium salt, and improving the primary cook’s knowledge and skills through materials and lectures. The baseline survey shows that using salt-restricting spoons is linked to lower sodium intake and BP [17]. As a pivotal strategy for reducing BP, low-sodium salt is instrumental in both the reformulation of processed foods and the curtailment of salt intake in households [18]. However, it carries the potential risk of exacerbating hyperkalemia, particularly among individuals with conditions that compromise potassium excretion, such as chronic kidney disease. The RCT research conducted in China further demonstrates the extensive application potential of low-sodium salt [19]. Concurrently, Chinese researchers have formulated guidelines for the utilization and promotion of low-sodium salt within the country, which are designed to offer expert recommendations on the promotion and application of low-sodium salt in China [20]. The findings of this study also indicate that the awareness and utilization of low-sodium salt are indeed beneficial for BP control.

A reduction in sodium intake at the population level is correlated with a corresponding decrease in BP. Our findings align with those from other studies, especially regarding the more significant reduction in SBP. For example, a meta-analysis has shown that, for every 1725 mg decrease in daily sodium intake, there is a corresponding reduction in SBP by 4.1 mmHg [21,22]. However, some studies present divergent results. In the Shandong–Ministry of Health Action on Salt and Hypertension (SMASH) program, despite a certain decrease in salt intake, the reduction in both SBP and DBP was less pronounced [14]. This inconsistency might be due to the stronger correlation of DBP with environmental and risk factors. Our study also observed a slight but statistically significant increase in SBP in the reference group (*p* < 0.05), which suggests the natural progression of BP increase with age. The change in DBP was not statistically significant. After the intervention, the BP in the intervention group exhibited a decrease, indicating that the measures implemented were effective in reducing BP, even when considering age-related and environmental factors.

Boosting potassium intake while cutting sodium is crucial for lowering BP, especially in high-sodium consumers who benefit more from potassium’s BP-lowering effect [5,18,23]. Notably, research has shown that replacing regular table salt with healthier alternatives can result in a decreased incidence of hypertension among elderly Chinese individuals with normal BP [19]. This dietary shift does not seem to elevate the risk of developing hypotension. Our study indicates that participants in the intervention group increased their potassium intake following the intervention, a factor associated with the observed reduction in blood pressure across the population. The ratio of sodium to potassium, a well-established indicator for assessing the effectiveness of intervention strategies, has been significantly high in China, nearly reaching five. The reduction rate of 1.2 achieved in this study is indicative of the substantial positive effects of our comprehensive interventions, which were designed to decrease sodium consumption and enhance potassium supplementation.

In this study, sodium intake in the intervention area did not exhibit a significant change before and after the intervention. A nationally representative cross-sectional survey conducted in 2015 revealed that the overall weighted average for 24-hUNa excretion for adults in China was 4121 mg per day [13]. Our study measured an overall average 24-hUNa level of 3849.5 mg/day, with the intervention group’s baseline level at 3701.0 mg/day, both below the national average. Concurrently, the average sodium intake for American adults during the same period was 3608 mg per day [24]. This comparison underscores the difficulty in reducing salt intake in this region, and suggests that the potential for further reduction may be limited. Furthermore, the process of adopting sodium reduction behaviors and the associated behavioral changes among individuals with a high-sodium diet is often iterative and variable. It is likely that, in the middle and late stages of the intervention, the emphasis on salt reduction interventions may need to be intensified. Moreover, this study noted a pronounced change in sodium salt levels among individuals with normal BP, in contrast to the more noticeable changes in BP among those with hypertension. This discrepancy may be due to hypertensive patients’ reliance on antihypertensive medications and a disregard for dietary factors. Adhering to medication is perceived as more straightforward than reducing sodium intake, a critical yet often overlooked strategy for lowering blood pressure. A significant reduction in sodium intake over a brief period was associated with positive blood-pressure-lowering effects; however, such outcomes may be more readily achievable in community trials [25].

Previous studies have shown that acquiring comprehensive knowledge and adopting dynamic measures can markedly decrease the likelihood of developing hypertension, including for individuals who have already reached a prehypertensive state [26]. There exists a significant opportunity to elevate the Chinese population’s awareness and practices concerning salt consumption. This study reveals a strong linkage, indicating that an informed and proactive stance on KABs can effectively decrease sodium levels and augment potassium intake, notably the latter, which is essential in preventing the advancement of hypertension. The value of beliefs within the KAB framework deserves full recognition and enhancement, on par with the advancement of knowledge and the encouragement of active behaviors [27]. This research provides vital evidence and insights, supporting China’s efforts to reduce salt intake and prevent hypertension.

This study represents a large-scale population intervention study focused on key outcome indicators: BP and sodium intake. The 24-hUNa measurement is regarded as a precise method for assessing sodium consumption. Additionally, this study implemented standardized protocols for urine collection, measurement, and inclusion criteria, along with rigorous on-site quality control. In this research, a reference group was established to assess the efficacy of the intervention strategies. As secondary outcomes, KABs related to salt intake were examined, which can effectively elucidate the effectiveness of the intervention and any changes in the primary outcomes. The findings of this study demonstrate that a multi-sectoral approach, combined with extensive public awareness initiatives and precisely targeted interventions, can significantly reduce sodium intake and blood pressure. These results have important implications for public health policy. The strategies employed in this study, such as distributing salt restriction spoons, promoting low-sodium salt, and enhancing community-based education, can be replicated and scaled up in other regions. Furthermore, the establishment of enduring systems and mechanisms for salt reduction can contribute to long-term sustainability and broader population health improvements.

However, our study has certain limitations. Due to its high degree of randomness, the random sequence method may not effectively control known confounding factors, thereby affecting the accuracy and reliability of the study results. In both surveys, a single instance of 24 h urine was collected instead of multiple samples over different days. Due to variations in sodium intake influenced by diet, exercise, and other factors, the 24-hUNa measurements could be subject to instability [28]. To capture these daily variations accurately, multiple urine collections over several days are essential [29]. Research exploring the potential of single-day measurements, combined with external variables, to predict habitual salt intake distribution appears to be a promising approach [30]. Furthermore, the two-year duration of the intervention may have been influenced by other public health services and disease-related changes that could impact behaviors, thereby affecting BP and the levels of urine sodium. The absence of monitoring data for sodium and potassium excretion, as well as blood pressure, throughout the intervention period poses challenges in interpreting the research findings and distilling the intervention strategies.

## 5. Conclusions

The SRHPP program demonstrates that multi-sectoral collaboration, extensive public awareness campaigns, and targeted intervention strategies can effectively lead to an increase in 24-hUKa excretion and a decrease in 24-hUNa excretion and BP. Concurrently, there is a significant enhancement in the level of KABs related to salt intake. This offers valuable insights and inspiration for developing population-based salt reduction interventions aimed at the prevention and management of cardiovascular and cerebrovascular diseases.

## Figures and Tables

**Table 1:** Characteristics of participants in intervention and reference groups between preintervention and postintervention surveys ^a^.

Characteristic	Intervention			Reference		
	Preintervention	Postintervention	*p* Value ^b^	Preintervention	Postintervention	*p* Value ^b^
Age, years	46.5 (13.7)	50.2 (13.8)	<0.001 *	46.0 (13.8)	49.8 (13.7)	<0.001 *
Sex			1.00			1.00
Female	3001 (50.0%)	2518 (52.1%)		765 (50.7%)	605 (53.4%)	
Male	3002 (50.0%)	2317 (47.9%)		744 (49.3%)	528 (46.6%)	
Education			1.00			1.00
Primary school or lower	1893 (31.6%)	1589 (33.4%)		388 (25.7%)	338 (28.3%)	
Junior high school	2848 (47.5%)	2244 (47.1%)		760 (50.4%)	580 (48.5%)	
College or higher	1259 (21.0%)	928 (19.5%)		361 (23.9%)	278 (23.2%)	
Cigarette smoking			<0.001 *			0.26
Never smoked	4261 (71.0%)	3699 (76.9%)		1111 (73.6%)	908 (75.8%)	
Former smokers	266 (4.4%)	220 (4.6%)		46 (3.0%)	55 (4.6%)	
Current smokers	1473 (24.6%)	893 (18.6%)		352 (23.3%)	235 (19.6%)	
Alcohol use status	1925 (32.1%)	1076 (22.4%)	<0.001 *	549 (36.4%)	352 (29.4%)	<0.001 *
Physical activity	2418 (40.3%)	2334 (48.5%)	<0.001 *	602 (39.9%)	517 (43.2%)	0.05
Use of antihypertensive medications	780 (13.0%)	979 (20.3%)	<0.001 *	181 (12.0%)	239 (20.6%)	<0.001 *
BMI, kg/m^2^			0.043 *			<0.001 *
Low weight	231 (3.8%)	187 (3.9%)		53 (3.5%)	26 (2.2%)	
Normal	3048 (50.1%)	2429 (50.4%)		726 (48.0%)	528 (45.5%)	
Overweight	2044 (33.6%)	1655 (34.3%)		546 (36.1%)	426 (36.7%)	
Obese	679 (11.2%)	551 (11.4%)		184 (12.2%)	181 (15.6%)	
Mean	24.0 (3.3)	23.9 (3.4)	0.05	24.2 (3.4)	24.5 (3.5)	<0.001 *
SBP, mmHg	131.2 (19.2)	129.8(18.0)	<0.001 *	127.0 (18.7)	127.8 (17.8)	0.049 *
DBP, mmHg	80.8 (10.8)	78.9 (10.2)	<0.001 *	80.5 (10.9)	80.2 (10.7)	0.47
BP status			0.69			<0.001 *
Normal	1677 (27.9%)	1282 (26.5%)		517 (34.3%)	339 (29.0%)	
Prehypertension	2154 (35.9%)	1678 (34.7%)		511 (33.9%)	376 (32.2%)	
Hypertension	2172 (36.2%)	1869 (38.7%)		481 (31.9%)	453 (38.8%)	
Diabetes mellitus	428 (7.1%)	529 (10.9%)	<0.001 *	137 (9.1%)	150 (12.4%)	<0.001 *
24-hUNa excretion, mg/24 h	3701.0 (1641.8)	3615.1 (1788.9)	0.24	4227.0 (1473.7)	4149.8 (1770.4)	0.58
24-hUKa excretion, mg/24 h	1441.3 (681.9)	1676.9 (931.4)	<0.001 *	1726.6 (671.5)	2017.2 (783.5)	<0.001 *
Sodium-to-potassium ratio	4.8 (2.2)	3.6 (1.7)	<0.001 *	4.6 (2.2)	4.2 (1.9)	0.031 *

^a^ Data are mean (SD) n (%) unless otherwise stated. ^b^ *p* value for difference between preintervention and postintervention based on paired-sample *t* test for continuous variable and χ^2^ test for categorical variable. * *p* < 0.05. Abbreviations: BMI: body mass index; BP: blood pressure; DBP: diastolic blood pressure; SBP: systolic blood pressure; SD: standard deviation; 24-hUKa: 24 h urinary potassium; 24-hUNa: 24 h urinary sodium.

**Table 2:** Adjusted net systolic and diastolic blood pressure and 24 h urinary sodium and potassium excretion between intervention and reference group in SRHPP from 2017 to 2021.

	Mean Change (95%CI) ^a^				Net Difference (95%CI) ^b^	*p* Value	Adjusted Net Difference (95%CI) ^c^	*p* Value
	Intervention	*p* Value	Reference	*p* Value				
Change in SBP, mmHg	1.4 (0.9 to 1.8)	<0.001 *	−0.9 (−1.8 to −0.0)	0.049 *	2.3 (1.3 to 3.3)	<0.001 *	1.3 (0.5 to 2.1)	<0.001 *
Change in DBP, mmHg	1.8 (1.5 to 2.1)	<0.001 *	0.2 (−0.4 to 0.8)	0.47	1.6 (0.9 to 2.2)	<0.001 *	1.4 (0.9 to 2.0)	<0.001 *
Change in 24−hUNa excretion, mg	85.9 (−57.7 to 229.5)	0.24	77.3 (−195.9 to 350.4)	0.58	8.6 (−309.2 to 326.4)	0.96	394.1 (133.2 to 655.0)	0.003 *
Change in 24−hUKa excretion, mg	−235.5 (−304.0 to −167.1)	<0.001 *	−290.6 (−420.6 to −160.5)	<0.001 *	55.0 (−96.7 to 206.7)	0.48	240.9 (108.2 to −373.6)	<0.001 *

^a^ Change refers to the difference from baseline to terminal investigation. ^b^ Unadjusted net difference. ^c^ Adjusted for age, sex, education, history of antihypertensive treatment, baseline body mass index, systolic (or diastolic) blood pressure, and 24-hUNa, 24-hUKa excretion. * *p* < 0.05. Abbreviations: CI: confidence interval; DBP: diastolic blood pressure; SBP: systolic blood pressure; 24-hUKa: 24 h urinary potassium; 24-hUNa: 24 h urinary sodium.

**Table 3:** Knowledge, attitudes, and behaviors associated with sodium reduction in SRHPP from 2017 to 2021.

	Proportion (%)						Net Difference (%) ^a^	Adjusted OR (95%CI) ^b^	*p* Value
	Intervention			Reference					
	Preintervention (d1)	Postintervention (d2)	*p* Value	Preintervention (d3)	Postintervention (d4)	*p* Value			
**Knowledge**									
Know the diagnostic criteria of hypertension	50.3	65.1	<0.001 *	64.8	68.4	0.13	11.1	1.45 (1.33 to 1.59)	<0.001 *
Know the hazards of hypertension	72.9	84.9	<0.001 *	81.1	87.1	<0.001 *	6.0	1.36 (1.20 to 1.53)	<0.001 *
Know the risk factors of hypertension	78.2	88.6	<0.001 *	84.2	90.0	<0.001 *	4.6	1.27 (1.11 to 1.45)	<0.001 *
Know less than 6 g salt per day	35.2	57.5	<0.001 *	53.7	59.4	0.01 *	16.6	1.57 (1.43 to 1.72)	<0.001 *
Know that eating less salt lowers blood pressure	63.3	79.8	<0.001 *	75.4	82.6	<0.001 *	9.3	1.48 (1.33 to 1.64)	<0.001 *
Know the hazards of high salt	69.3	85.4	<0.001 *	78.3	87.7	<0.001 *	6.6	1.37 (1.22 to 1.54)	<0.001 *
Know what kind of people should eat a low-salt diet	78.0	91.8	<0.001 *	84.8	91.7	<0.001 *	6.8	1.28 (1.12 to 1.46)	<0.001 *
Know how to use salt restriction spoon correctly	8.1	32.3	<0.001 *	11.5	21.0	<0.001 *	14.7	1.32 (1.17 to 1.49)	<0.001 *
Know low-sodium salt	29.0	44.6	<0.001 *	34.1	43.5	<0.001 *	6.1	1.02 (0.93 to 1.12)	0.61
Know that low-sodium salt helps control blood pressure	19.6	35.9	<0.001 *	25.6	36.0	<0.001 *	5.9	1.10 (1.00 to 1.22)	0.049 *
**Attitude**									
Evaluate whether low-salt diet affects taste of food			<0.001 *			0.87		1.35 (1.24 to 1.47)	<0.001 *
Great influence	11.4	9.8		12.5	12.6		−1.7		
Has some influence, but can accept	62.1	58.8		65.9	65.8		−3.1		
No effect	26.5	31.3		21.6	21.6		4.8		
Approve that low-salt diet should be promoted among the crowd	86.4	93.2	<0.001 *	89.9	92.7	0.04 *	3.9	1.14 (0.98 to 1.32)	0.09
Approve of low-salt diet	87.8	91.8	<0.001 *	89.7	90.5	0.75	3.2	1.01 (0.88 to 1.16)	0.90
Approve of the nutrition labeling of prepackaged food	74.9	77.3	<0.001 *	75.8	76.0	0.57	2.2	1.11 (1.00 to 1.23)	0.06
Approve that the nutrition labeling of prepackaged food will help to choose low-salt diet	72.3	76.3	<0.001 *	74.0	75.4	0.09	2.7	1.06 (0.96 to 1.17)	0.28
**Behavior**									
Self-assessment salt level			<0.001 *			0.26		1.74 (1.61 to 1.89)	<0.001 *
Not much	31.3	35.5		17.2	18.6		2.8		
Moderate	50.2	53.9		63.7	64.2		3.2		
Excessive	18.5	10.5		19.2	17.2		−6.1		
Received publicity or education on low-salt diet	45.8	64.2	<0.001 *	61.7	72.2	<0.001 *	7.9	1.68 (1.53 to 1.83)	<0.001 *
Once promoted low-salt diet knowledge to people	68.0	57.9	<0.001 *	72.3	63.1	0.33	−0.9	1.17 (1.06 to 1.30)	0.003 *
Pay attention to the nutrition label of prepackaged food	20.6	31.0	<0.001 *	28.3	32.3	0.10	6.4	1.20 (1.09 to 1.33)	<0.001 *
Plan to reduce salt	78.0	90.3	<0.001 *	78.2	87.3	<0.001 *	3.2	1.14 (1.02 to 1.28)	0.02 *
Take initiative to reduce salt	58.2	70.6	<0.001 *	58.6	66.2	<0.001 *	4.8	1.12 (1.02 to 1.22)	0.02 *
Using or used salt restriction spoon	11.1	39.0	<0.001 *	15.3	24.0	<0.001 *	19.1	1.37 (1.22 to 1.53)	<0.001 *
Using salt restriction spoon correctly	5.1	26.0	<0.001 *	7.4	16.5	<0.001 *	11.8	1.36 (1.19 to 1.56)	<0.001 *
Using or used low-sodium salt	15.0	21.8	<0.001 *	18.5	21.0	0.19	4.3	1.04 (0.93 to 1.17)	0.45

^a^ Net difference means (d2–d1) minus (d4–d3). ^b^ Adjusted for age, sex, education, history of antihypertensive treatment, baseline body mass index, systolic (or diastolic) blood pressure, and 24-hUNa and 24-hUKa excretion. * *p* < 0.05. Abbreviations: CI: confidence interval; OR: odds ratio; 24-hUKa: 24 h urinary potassium; 24-hUNa: 24 h urinary sodium.

## Data Availability

The datasets utilized in this study are not accessible to the public due to intellectual property restrictions; however, they may be obtained from the corresponding author upon a legitimate request.

## Supplementary Materials

The following supporting information can be downloaded at: https://www.mdpi.com/article/10.3390/nu17050893/s1, Figure S1: Flow of participants through the SRHPP trial; Table S1: Questionnaire about knowledge, attitudes and behaviors related to salt, SRHPP 2017–2021; Table S2: Adjusted net systolic and diastolic blood pressure, 24-h urinary sodium and potassium excretion between intervention and reference group across various hypertension conditions in SRHPP from 2017 to 2021.

## Abbreviations

BMI	body mass index;
BP	blood pressure;
CDC	Center for Disease Control and Prevention;
CI	confidence interval;
CVD	cardiovascular diseases;
DBP	diastolic blood pressure;
KABs	knowledge, attitudes, and behaviors;
OR	odds ratio;
RCT	randomized controlled trial;
SBP	systolic blood pressure;
SD	standard deviation;
SMU	second morning urine;
SRHPP	Salt Reduction and Hypertension Prevention Project;
SU	spot urine;
WHO	World Health Organization;
24-hUKa	24 h urinary potassium;
24-hUNa	24 h urinary sodium.
