# Supplementary material for: Effect of Salt Reduction Interventions in Lowering Blood Pressure and Salt Intake in Zhejiang Province, China, 2017–2021: A Randomized Controlled Trial
Source: Nutrients. 2025 Mar 3;17(5):893. doi: 10.3390/nu17050893 (PMC11901917; doi:10.3390/nu17050893)
Supplement: Supplementary file 1 [file nutrients-17-00893-s001.zip › nutrients-3496644-supplementary.pdf]

*Supplementary Material*

# Effect of salt reduction interventions in lowering blood pressure and salt intake in Zhejiang Province, China, 2017-2021： a randomized controlled trial

**The first author: Xiaofu Du**

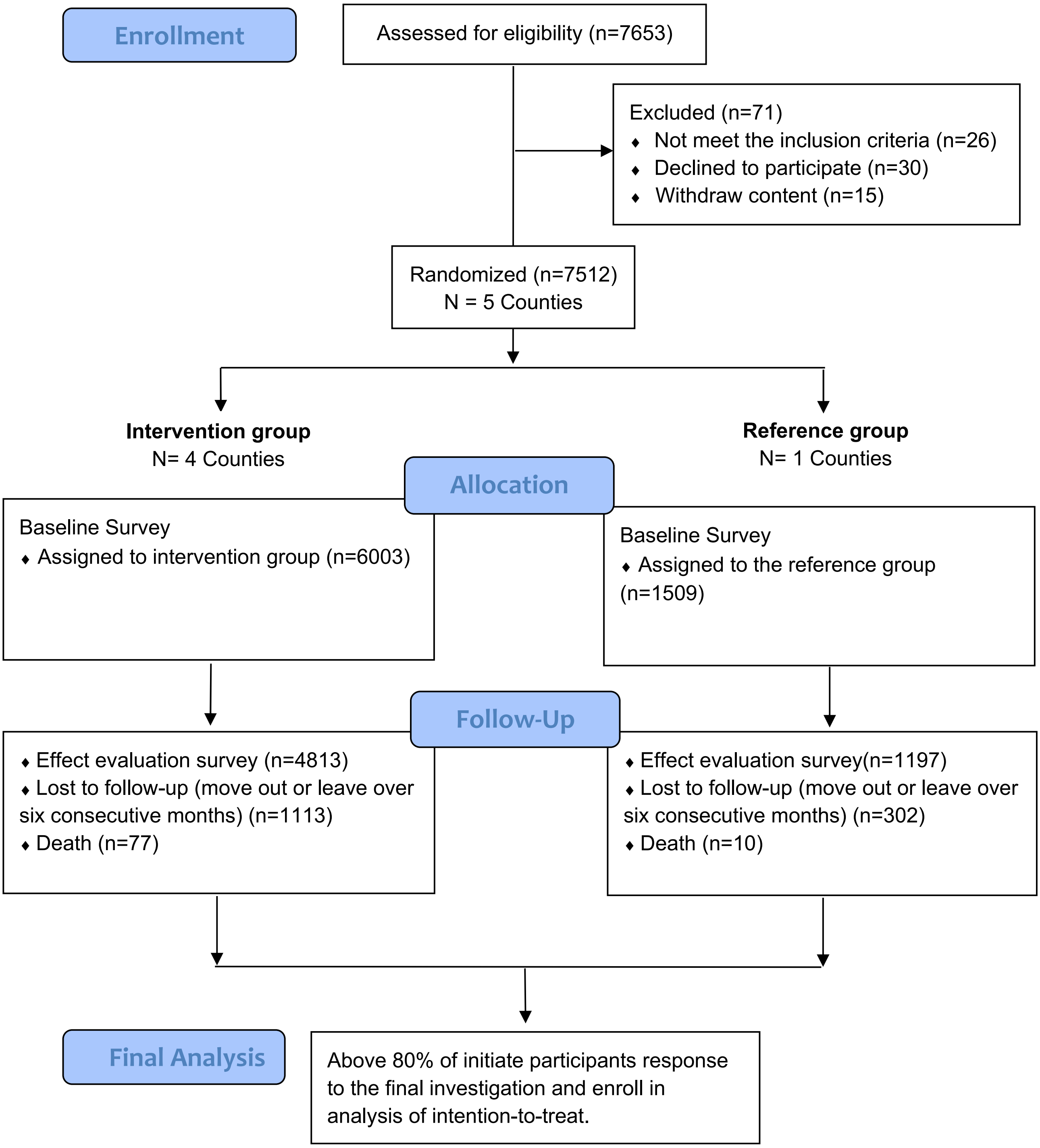


**Supplemental Figure S1.** Flow of participants through the SRHPP trial

**Supplemental Table S1.** Questionnaire about knowledge, attitudes and behaviors related to salt, SRHPP 2017-2021.

1. Do you know how much blood pressure in adults can be diagnosed as hypertension?

(1) 140/90mmHg

(2) 130/80 mmHg

(3) 120/80 mmHg

(9) I don’t know

2. Do you know what diseases hypertension can cause (multiple choices)?

(1) Stroke

(2) Coronary heart disease

(3) Kidney disease

(4) Hypertensive heart disease

(5) Eye diseases

(9) I don’t know

3. Do you know which factors are related to the prevalence of hypertension (multiple choices)?

(1) Overweight or obese

(2) Long-term excessive drinking

(3) Long-term high-salt diet

(4) Have a family genetic history

(5) Hyperglycemia and hyperlipidemia

(6) Age

(7) Stress

(9) I don’t know

4. Do you know that adults should not eat more than a few grams of salt per person per day?

(1) 2g

(2) 6g

(3) 9g

(4) 12g

(9) I don’t know

5. Do you know that eating less salt helps lower blood pressure?

(1) Know

(2) I don’t know

6. Do you know what diseases may result from eating too much salt (multiple choices)?

(1) Hypertension

(2) Stroke

(3) Myocardial infarction

(4) Kidney disease

(5) Gastric cancer

(6) Osteoporosis

(9) I don’t know

7. Do you think you eat too much salt?

(1) Not much

(2) Moderate

(3) Too much

8. Do you plan to eat less salt after knowing the dangers of eating too much salt?

(1) Intend to

(2) Not intend to

9. Has your family ever used a salt-restriction spoon?

(1) Used

(2) Not used

10. Does your family know how to use salt-restriction spoon?

(1) Know

(2) I don't know

11. Can your family use the salt-restriction spoon correctly according to the requirements?

(1) Yes

(2) No

12. Do you think low-salt diet should be promoted among the crowd?

(1) Should

(2) Should not

13. Have you ever been promoted or educated on a low-salt diet?

(1) Accepted

(2) Not accepted

14. Have you ever promoted the knowledge of low-salt diet to the people around you?

(1) Promoted

(2) Not promoted

15. Do you think a low-salt diet affects the taste of food?

(1) Great influence

(2) Has a certain influence, but can accept

(3) No effect

16. What kind of people do you think should pay special attention to low-salt diet (multiple choices)?

(1) Hypertensive patients

(2) Patients with stroke

(3) Patients with coronary heart disease

(9) I don't know

17. Have you paid attention to the salt / sodium content of the food when purchasing processed food?

(1) Followed

(2) Not followed

18. Do you think processed foods should be labeled with the salt / sodium content of the product?

(1) Should

(2) Should not

19. Do you think that labeling the salt / sodium content of processed foods will help you choose low-salt foods?

(1) Yes

(2) No

20. What is your attitude towards a low-salt diet?

(1) For

(2) Against

21. Have you taken active salt reduction measures yourself?

(1) Yes

(2) No

22. Have you heard of low-sodium salt (alternative salt)?

(1) Yes

(2) No

23. Do you know that low-sodium salt helps control blood pressure compared to regular table salt?

(1) Know

(2) I don't know

24. Have you used low-sodium salt?

(1) Used

(2) Not used

**Supplemental Table S2.** Adjusted net systolic and diastolic blood pressure, 24-h urinary sodium and potassium excretion between intervention and reference group across various hypertension conditions in SRHPP from 2017 to 2021

| | Mean change (95% CI) [a] | | | | Net difference (95% CI) [b] | *P* value | Adjusted net difference (95%CI) [c] | *P* value |
|---|---|---|---|---|---|---|---|---|
| | Intervention | *P* value | Reference | *P* value | | | | |
| Change in SBP, mm Hg | 1.4 (0.9 to 1.8) | <0.001* | -0.9 (-1.8 to -0.0) | 0.049* | 2.3 (1.3 to 3.3) | <0.001* | 1.3 (0.5 to 2.1) | <0.001* |
| Normotensive | -6.0 (-6.7 to -5.2) | <0.001* | -6.6 (-7.9 to -5.3) | <0.001* | 0.6 (-0.8 to 2.1) | 0.39 | 0.6 (-0.3 to 1.5) | 0.16 |
| Prehypertensive | 0.4 (-0.2 to 1.1) | 0.16 | -1.2 (-2.5 to 0.0) | 0.05 | 1.7 (0.3 to 3.1) | 0.02* | 1.2 (0.3 to 2.1) | 0.008* |
| Hypertensive | 7.1 (6.3 to 7.9) | <0.001* | 5.2 (3.4 to 7.0) | <0.001* | 1.9 (-0.0 to 3.9) | 0.05 | 2.0 (0.9 to 3.1) | <0.001* |
| Change in DBP, mm Hg | 1.8 (1.5 to 2.1) | <0.001* | 0.2 (-0.4 to 0.8) | 0.47 | 1.6 (0.9 to 2.2) | <0.001* | 1.4 (0.9 to 2.0) | <0.001* |
| Normotensive | -2.5 (-2.9 to -2.0) | <0.001* | -3.3 (-4.2 to -2.5) | <0.001* | 0.8 (-0.1 to 1.8) | 0.08 | 0.9 (0.0 to 1.8) | 0.04* |
| Prehypertensive | 1.0 (0.6 to 1.4) | <0.001* | 0.0 (-0.9 to 0.9) | 1.00 | 1.0 (0.1 to 2.0) | 0.03* | 1.4 (0.5 to 2.2) | 0.002* |
| Hypertensive | 5.3 (4.8 to 5.8) | <0.001* | 4.0 (2.9 to 5.1) | <0.001* | 1.4 (0.2 to 2.5) | 0.02* | 2.2 (1.2 to 3.2) | <0.001* |
| Change in 24-hUNa excretion, mg | 85.9 (-57.7 to 229.5) | 0.24 | 77.3 (-195.9 to 350.4) | 0.58 | 8.6 (-309.2 to 326.4) | 0.96 | 394.1 (133.2 to 655.0) | 0.003* |
| Normotensive | 307.4 (14.3 to 600.5) | 0.04* | 102.3 (-396.4 to 601.1) | <0.001* | 205.1 (-389.5 to 799.6) | 0.50 | 701.4 (233.8 to 1168.9) | 0.003* |
| Prehypertensive | -182.0 (-426.5 to | 0.14 | -27.0 (-454.0 to | 0.90 | -155.0 (-686.5 to | 0.57 | 290.9 (-185.0 to | 0.23 |

| | | | | | | | | |
|---|---|---|---|---|---|---|---|---|
| | 62.4) | | 400.0) | | 376.4) | | 766.9) | |
| Hypertensive | 154.2 (-67.2 to 375.6) | 0.17 | 153.0 (-354.6 to 660.6) | 0.55 | 1.2 (-533.2 to 535.5) | 1.00 | 255.1 (-167.8 to 678.0) | 0.24 |
| Change in 24-hUKa excretion, mg | -235.5 (-304.0 to -167.1) | <0.001* | -290.6 (-420.6 to -160.5) | <0.001* | 55.0 (-96.7 to 206.7) | 0.48 | 240.9 (108.2 to -373.6) | <0.001* |
| Normotensive | -225.9 (-366.5 to -85.3) | 0.002* | -268.5 (-500.4 to -36.5) | <0.001* | 42.6 (-240.8 to 326.0) | 0.77 | 244.6 (3.1 to 486.0) | 0.047* |
| Prehypertensive | -207.1 (-334.0 to -80.1) | 0.001* | -367.9 (-581.0 to -154.8) | <0.001* | 160.9 (-115.4 to 437.1) | 0.25 | 228.3 (-13.6 to 470.2) | 0.06 |
| Hypertensive | -265.7 (-363.5 to -167.9) | <0.001* | -237.9 (-476.2 to 0.5) | 0.05 | -27.8 (-266.0 to 210.3) | 0.82 | 246.9 (30.8 to 463.0) | 0.03* |

[a] Change refers to the difference from baseline to terminal investigation

[b] Unadjusted net difference.

[c] Adjusted for age, sex, education, history of antihypertensive treatment, baseline body-mass index, systolic (or diastolic) blood pressure and 24-hUNa excretion, 24-hUKa excretion.

* $P < 0.05$

Abbreviations: CI: confidence interval; DBP: diastolic blood pressure; SBP: systolic blood pressure. 24-hUKa: 24-hour urinary potassium; 24-hUNa: 24-hour urinary sodium.
